# Multi-Temporal InSAR Analysis for Monitoring Ground Deformation in Amorgos Island, Greece †

**DOI:** 10.3390/s20020338

**Published:** 2020-01-07

**Authors:** Stavroula Alatza, Ioannis Papoutsis, Demitris Paradissis, Charalampos Kontoes, Gerassimos A. Papadopoulos

**Affiliations:** 1 Higher Geodesy Laboratory and Dionysos Satellite Observatory, School of Rural and Surveying Engineering, National Technical University of Athens, 9 Iroon Polytechniou str, 15780 Zographos, Greece; dempar@central.ntua.gr; 2 Institute for Astronomy, Astrophysics, Space Applications & Remote Sensing, National Observatory of Athens, Vas. Pavlou & I. Metaxa str, 15236 Penteli, Greece; ipapoutsis@noa.gr (I.P.); kontoes@noa.gr (C.K.); 3 International Society for the Prevention & Mitigation of Natural Hazards, 10681 Athens, Greece; gerassimospapadopoulos2@gmail.com

**Keywords:** SAR interferometry, amorgos 1956 earthquake, Sentinel-1, timeseries analysis, LOS decomposition, ground deformation

## Abstract

Radar Interferometry is a widely used method for estimating ground deformation, as it provides precision to a few millimeters to centimeters, and at the same time, a wide spatial coverage of the study area. On 9 July 1956, one of the strongest earthquakes of the 20th century in the area of the South Aegean, occurred in Amorgos, with a magnitude of Mw = 7.7. The objective of this research is to map ground deformation in Amorgos island, using InSAR techniques. We conducted a multi-temporal analysis of all available data from 2003 to 2019 by exploiting historical ENVISAT SAR imagery, as well as the dense archive of Sentinel-1 SLC imagery. Persistent Scatterer Interferometry (PS) and Small Baseline Subset (SBAS) methods were implemented. Results of both data-sets indicate a small-scale deformation on the island. A multi-track analysis was implemented on Sentinel-1 data to decompose the line of sight velocities to vertical and horizontal. The central south coast is experiencing horizontal movement, while uplift of a maximum value of 5 mm/y is observed in the southeastern coast. The combination of the good spatial coverage achievable via InSAR, with GPS measurements, is suggested an important tool for the seamless monitoring of Amorgos island towards tectonic hazard estimation.

## 1. Introduction

Greece is situated on the convergence boundaries between the Eurasian and the African lithospheric plates, and therefore is one of the most seismogenic areas in the world. On 9 July 1956, Greece experienced the strongest shallow earthquake in the last 100 years or so. Its moment-magnitude was measured to be equal to Mw = 7.7, while the focal depth was estimated at 20 km [1]. This large earthquake ruptured the area of the submarine trough between Amorgos, Santorini, and Astypalea in the Cyclades island complex, South Aegean Sea [2].

The earthquake produced a large tsunami. Yet, it is not clear if it was generated by co-seismic fault displacement or by submarine sediment slumping [3,4,5,6,7]. Extensive destruction was caused by the 1956 earthquake particularly in Santorini and Amorgos, but also in the surrounding islands. The seismic event caused a death toll of 53 people, while 100 people were injured, 529 buildings were destroyed, and more than 3200 buildings were severely damaged (e.g., [3,8]). The tsunami wave inundated coastal zones all over the southern Aegean Sea region, resulting in four victims and some damage [3].

In this study we present small-scale ground displacements measured on Amorgos island through geodetic observations collected from a time period extended to more than a decade. To produce ground velocity maps for the island of Amorgos, we conducted a multi-temporal analysis of Synthetic Aperture Radar (SAR) imagery, from 2003 to 2019, using Persistent Scatterer Interferometry (PS) [9] and Small Baseline Subset (SBAS) [10] techniques, through a coherent combination of the two products [11,12]. A decomposition of the line of sight velocities to vertical and horizontal components was performed, combining measurements from ascending and descending imaging geometries for the 2014–2019 period and an estimation of vertical velocities for the 2003–2010 period.

## 2. Tectonic Setting

**Figure 1:**
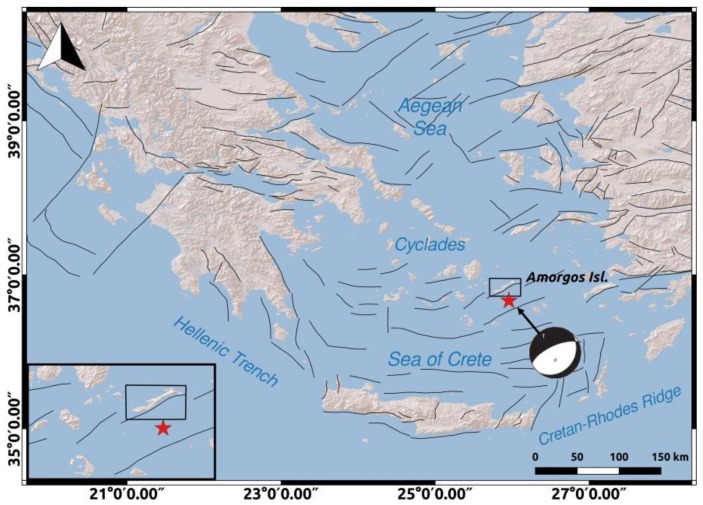
Fault sources in Greece as obtained by The European Database of Seismogenic Faults [14]. The epicenter of the earthquake as obtained by the International Seismological Center-Global Earthquake Model (ISC-GEM) Global Instrumental Earthquake Catalogue [1] is depicted with the red star and the focal mechanism of the earthquake is added based on [7]. Amorgos is depicted with the black rectangle. The inset image is a closer view of Amorgos, the faults located near the island and the epicenter of the 9 July 1956 Amorgos earthquake.

The area of Amorgos island is characterized by neotectonic normal faults [2,13]. Fault sources in Greece and specifically the faults near Amorgos island, as obtained by The European Database of Seismogenic Faults [14], are presented in Figure 1. There exists morphotectonic evidence for long-term tilting of the island, with uplift of the SE coast and subsidence of the NW coast dominated by normal faulting [15,16], and this result is consistent with evidence of seismic uplift in the area of Hozoviotissa and drowning of ancient remains in the NE coast [16].

The destructive earthquake and tsunami of 1956, with an epicenter located within the Santorini-Amorgos graben, resulted in great interest within the scientific community. Evidence from eye witnesses and marine flood deposits [3,4,7,17,18] suggest that the highest sea wave amplitudes hit the south coast of Amorgos (20–30 m) and the north coast of Astypalea (10–20 m). Lower wave amplitudes, with a maximum value of 4 m, were observed in the north coast of Crete, in the Dodekanese islands and the Turkish west coast, while wave amplitudes of up to 14.6 m were observed on the west coast of Folegandros island.

The northern part of the Santorini-Amorgos graben is characterized by shallow seismicity at depths of less than 15–20 km [19]. The northern graben southeast of Amorgos shows only low seismic activity [20,21]. Amorgos experienced uplift in the southern coast of about 30 cm and subsidence in the north coast [16], as a result of the July 1956 seismic event. The study of this seismic event and the subsequent post-seismic deformation is beyond doubt of special importance for better understanding of the South Aegean geodynamics and seismotectonics.

## 3. Data-Sets and Processing Strategy

**Figure 2:**
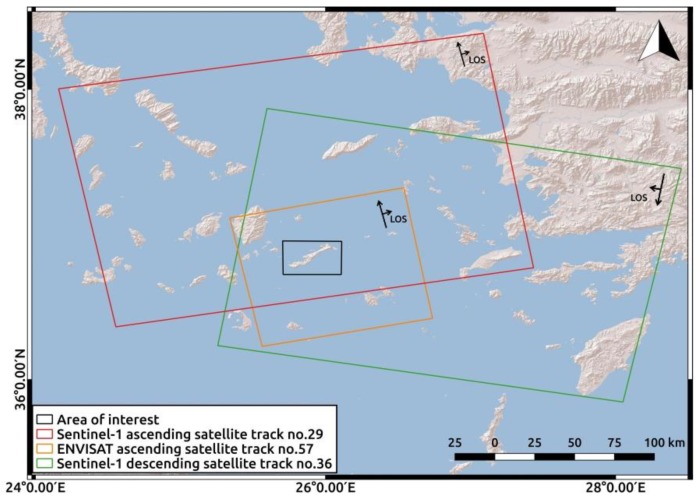
Map of the area of interest and the corresponding footprints of the satellite tracks of the data-sets.

Time-series analysis was performed on 24 ENVISAT SAR images from 2003 to 2010, as well as on 124 Sentinel-1 images from 2014 to 2019. The data-sets information is summarized in Table 1. Figure 2 illustrates the area of interest and the footprints of the satellite passes selected for Amorgos island. All scenes were provided by the European Space Agency (ESA). SNAP software (Sentinel Application Platform) [22] was used for the processing of Sentinel-1 imagery. Specifically, the formation and stacking of Sentinel-1 interferograms, was performed using the open-source package for SNAP-StaMPS integrated processing [23,24].

SNAP2StaMPS package uses the routines of the ESA SentiNel Application Platform (SNAP) to process Sentinel-1 images in an automated way, providing compatibility with StaMPS PSI as well. ROI_PAC software [25] (Repeat Orbit Interferometry Package), by California Institute of Technology and Jet Propulsion Laboratory and DORIS [26] (Delft object-oriented interferometric software version 4.06-beta2), developed by the Delft Institute of Earth Observation and Space Systems, were used for the processing of ENVISAT SAR data. The multi-temporal interferometry (MTI) was performed with StaMPS/MTI (Stanford Method for Persistent Scatterers—Multi-Temporal InSAR) [27,28], using PS and SBAS techniques, as well as the combination of the above methods. The open-source Toolbox for Reducing Atmospheric InSAR Noise (TRAIN) [29] was also employed by implementing a phase-based linear correction on interferograms produced using Sentinel-1 data.

### 3.1. The 2003–2010 Period

Twenty-four raw ENVISAT SAR acquisitions of ascending track 57 were processed for the period 2003 to 2010, from 2 May 2003 to 17 September 2010. The lack of an adequate number of scenes, in the descending track number 150 that covers the area of interest, led to the exclusion of this satellite pass from the estimation of ground displacements. An attempt to process seventeen available images, with a good spatial coverage of Amorgos island from the descending track number 150 was performed. The time span covered by this data set is from 2003 to 2010, with a four year time leap between the last scene of 2006 and the 2010 image. Therefore, the 2010 image was excluded from the processing owing to the generation of low SNR interferograms. Also, the poor image quality of several scenes and the low SNR of some interferograms led to the elimination of these problematic images, leading by extension to large temporal baselines. In our study case, the geomorphology of the island also introduced additional phase noise, which combined with the limited number of interferograms, compromised the accuracy of the estimated deformation signal. Thus, the descending imaging geometry of ENVISAT sensor was excluded from the time-series analysis. Estimating deformation rates before 2002, exploiting data of the ERS sensor was unfeasible. Available data-sets of ERS sensor, providing a good spatial coverage of Amorgos island, were very limited, including a maximum number of ten images per relative orbit and data-sets providing an adequate number of images for PS processing, were only partially covering the island, excluding a large NE part of the area of interest.

Initially, we focused the raw data to Single Look Complex images, using ROI_PAC software. In order to improve the density of the coherent pixels, we oversampled the SLC images by a factor of two in azimuth and range direction [30]. We used the orbital data of the Department of Earth Observation and space systems (DEOS) of the Delft University of Technology and ESA. Differential interferograms were formed with DORIS software. For the estimation of the topography effect, we used the SRTM DEM [31].

**Figure A1:**
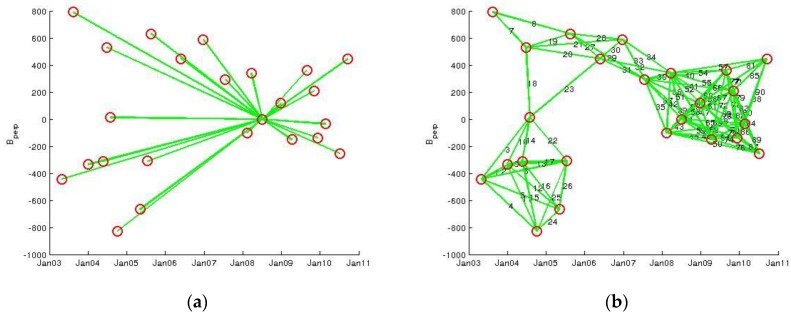
Baseline plots of ENVISAT data—Ascending track no.57, used for the time-series analysis in Amorgos island. The vertical axis represents the perpendicular baselines and the horizontal the dates of the ENVISAT images: (**a**) PS baseline plot; (**b**) SBAS baseline plot.

For the PS analysis, a master image is selected to form N-1 interferograms with all the remaining scenes. The PS technique identifies pixels with stable backscattering characteristics that are called persistent scatterers. In our study, the image that minimizes the temporal and perpendicular baselines is the scene of 4 July 2008. A baseline plot for the PS analysis is shown in Figure A1a of Appendix A. The SBAS technique, in StaMPS software version 4.1b, uses interferograms with the minimum Doppler, temporal, and perpendicular baselines. The formation of a dense, uniform network of interferograms is important for the SBAS analysis, as shown in the baseline plot in Figure A1b of Appendix A.

The maximum uncorrelated DEM error and the phase noise standard deviation for all pixel pairs are important parameters during the processing. To have a larger pixel sampling, considering the small interferometric stack, we selected a higher value of the default threshold (10 m) for the maximum topographic error, which leads to a higher mean γ value [27] of pixels that have random phase. The γ measure is a similar parameter to a measure of coherence [32]. A lower value for standard deviation threshold, at a subsequent processing step, contributed to the elimination of noisy pixels, and those selected due to signal contribution from neighboring ground resolution elements.

Additionally, the combined MTI processing was applied. During this step, pixels selected by both PS and SBAS methods are combined before the phase unwrapping step. The advantage of this step is the mitigation of the phase unwrapping errors, leading to a higher signal-to-noise ratio (SNR) and an improved spatial sampling.

### 3.2. The 2014–2019 Period

The Copernicus program succeeded the Envisat mission, which ended on 8 April 2012. The Sentinel-1 mission consists of a constellation of two C-band sensors, Sentinel-1A (S-1A) and Sentinel-1B (S-1B). The short revisit time (6 days), offers a consistent long-term remote sensing data archive and provides reliability on applications, requiring long time-series. The Interferometric Wide swath (IW) mode, employed in the present study, acquires data with a 250 km swath, with high spatial resolution of 5 × 20 m, and by using Terrain Observation with Progressive Scans SAR (TOPS) captures three sub-swaths in range-direction [33]. Each sub-swath is separated in nine bursts in azimuth direction.

For the 2014 to 2019 period, we performed Persistent Scatterer analysis on Sentinel-1 data-sets of both ascending and descending tracks. 54 images from 8 October 2014 to 6 September 2019 of descending track no.36, and 70 images from 19 October 2014 to 17 September 2019 of ascending track no.29, were selected and processed.

The preprocessing of Sentinel-1 SLC images, as already mentioned, was performed in SNAP software. In order to achieve a less time-consuming process and meet the requirements of Sentinel data-sets in computing storage and memory, we used SNAP’s TOPSAR-Split operator, to select the swath and the number of bursts that cover Amorgos island. Orbital information was used, to gain accurate satellite position and velocity information. Elevation Antenna Patterns correction was applied to Sentinel-1 acquisitions before March 2015, in order to compensate for the induced phase difference between the polarimetric channels.

**Figure A2:**
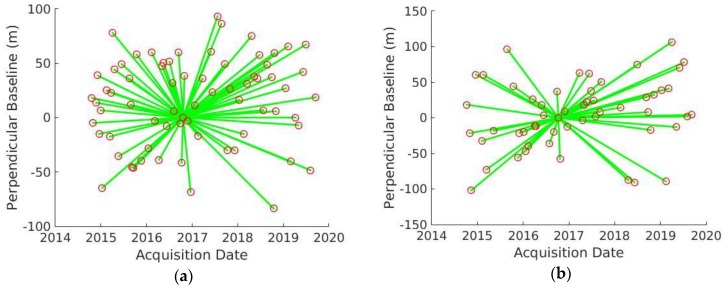
Baseline plots of Sentinel-1 data, used for the time-series analysis in Amorgos island. The vertical axis represents the perpendicular baselines and the horizontal the dates of Sentinel-1 SLC images: (**a**) PS baseline plot for the Ascending track no.29; (**b**) PS baseline plot for the Descending track no.36.

Taking benefit of the high temporal and spatial resolution, provided by the Sentinel-1 data, we were able to select and include in the PS processing the interferometric pairs, characterized by small baselines and high estimated coherence rates. The selection of the master image was based on the minimum temporal and perpendicular baseline thresholds. The maximum estimated coherence for the interferometric stacking was provided by the 9 October 2016 and the 20 October 2016 scenes for the descending track and the ascending track, respectively. The baseline plots for Sentinel-1 data are provided in Figure A2 of Appendix A.

All images are co-registered to the master image selected, using the back-geocoding operator. Before the interferogram formation, an additional processing of the bursts is necessary, due to the TOPS SAR mode characteristics. In Sentinel-1 TOPSAR, the imaged ground area of adjacent bursts will only marginally overlap in azimuth; therefore the deburst step is essential to solve pixel overlapping. Single master interferograms are formed, with subtraction of the flat-earth (reference) phase. The topographic phase was subtracted, using the SRTM DEM [31].

For the time-series analysis, we implemented the Stanford Method for Persistent Scatterers [27] as described in Section 3.1, referring to the 2003–2010 Envisat period.

## 4. Results

**Figure 3:**
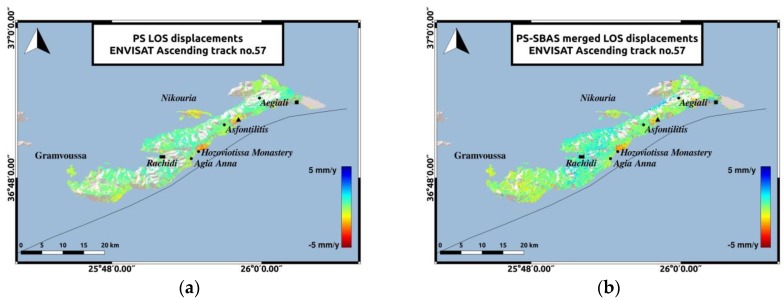
LOS displacements for ENVISAT SAR images-Ascending track no.57. The reference area near Rachidi is marked with the black rectangle. The black triangle represents the area between Asfontilitis and Aegiali sites and the black square represents the coast to the East of Aegiali. (**a**) PS LOS velocities; (**b**) PS-SBAS combination LOS velocities.

A flat residential area near Rachidi (Figure 3) was selected as a common reference area for all stacks, attempting to avoid areas with intense relief on the island or agricultural fields where water pumping can cause seasonal deformation signals. This area is assumed to be stable over time, in terms of deformation. In the absence of other geodetic observations on the island and accurate knowledge of the tectonic behavior of the reference area, we estimated the mean LOS velocity rates of all PS scatterers for all three stacks. The mean velocity rates are close to zero (0.63 mm/y for Sentinel-1 data of ascending track no.29, 0.69 mm/y for Sentinel-1 data of descending track no.36 and 0.63 mm/y for ENVISAT data), hinting that there is no bias in the estimated deformation rates.

Line of Sight velocities relative to this reference area were estimated for all data-sets. Although Persistent Scatterer Interferometry is very effective in analyzing deformation in urban areas, the StaMPS methodology exploits spatial correlation of interferogram’s phase to identify point targets in all terrains. In the Amorgos case, where most of the island consists of pastures and only 6% represents the residential area, the use of the StaMPS method ensured the identification of persistent scatterers. The PS processing of Sentinel-1 data resulted in 30,490 scatterers for the ascending track, 40,048 for the descending track and 19,899 PS scatterers for ENVISAT data.

In all stacks from 2003 to 2019, the LOS deformation rates are low, around 1mm/y in most parts of the island, which is close to the sensitivity limits of the PS technique as reported in [34,35,36]. However, in order to determine the absolute sensitivity for the specific dataset at hand, in-situ data must be used (e.g., cGPS measurements, leveling data), which are not available for Amorgos island.

The two islets of Nikouria and Gramvoussa were included in the InSAR analysis, assuming that from the coastline to the islets, the phase difference between two samples should be lower than π(|Δφ| = |φ_i_ − φ_i−1_| < π), as Amorgos island is under a very subtle deformation regime.

### 4.1. The 2003–2010 Period

The LOS displacements of the combined PS-SBAS analysis from 2003 to 2010 (Figure 3), indicate a small-scale displacement in most parts of the island and the two islets. Although the addition of the SBAS technique did not entail major alterations in the deformation pattern (Figure 3a), the combination of PS and SBAS results (Figure 3b), contributed to a better spatial sampling, with a total number of 38,525 permanent scatterers in the final deformation modeling.

**Figure 4:**
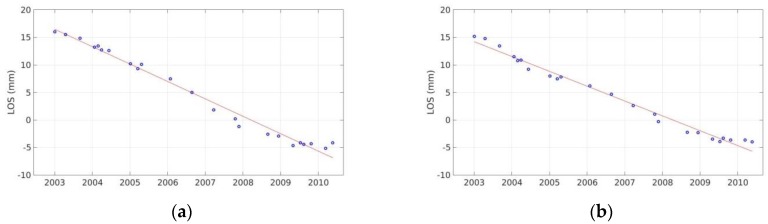
Time-series plots of scatterers included in a 100 m radius around the area of interest for ENVISAT data of ascending track no.57. (**a**) Time-series plot for Hozoviotissa Monastery area; (**b**) Time-series plot for the coast between Asfontilitis and Aegiali sites.

The LOS velocities estimated for the 2003–2010 time period (Figure 3) indicate motion of 5 mm/y, maximum rate, in a direction away from the satellite, near the Hozoviotissa Monastery region. To further investigate this observation, a time-series plot was generated for several scatterers within a region of 100 m radius, around the subsiding area (Figure 4a). Another area of interest is the coast between Asfontilitis and Aegiali sites, which is depicted in Figure 3 with the black triangle. During the time period investigated, the coast is also subsiding, but with lower velocity rates, not exceeding 4 mm/y. Figure 4a,b confirm the negative velocity rates observed in the LOS plots, for both subsiding areas.

### 4.2. The 2014–2019 Period

**Figure 5:**
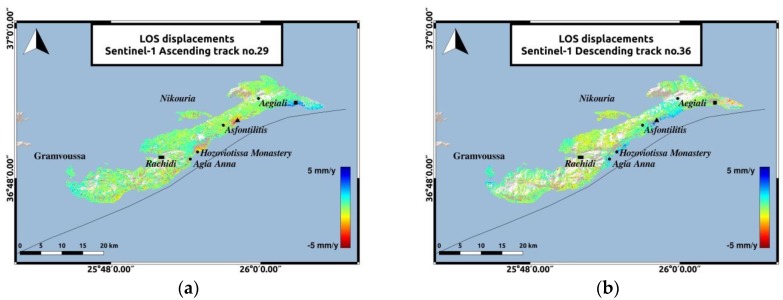
PS LOS velocities of Sentinel-1 data. The reference area near Rachidi is marked with the black rectangle. The black triangle represents the area between Asfontilitis and Aegiali sites and the black square represents the coast to the East of Aegiali. (**a**) PS LOS velocities for Sentinel-1 data of the ascending track no.29; (**b**) PS LOS velocities for Sentinel-1 data of the descending track no.36.

**Figure 6:**
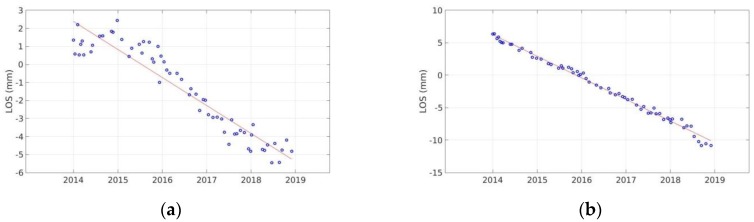
Time-series plots for scatterers included in a 100 m radius around the area of interest for Sentinel-1 data, of the ascending track no.29. (**a**) Time-series plot for Hozoviotissa Monastery area; (**b**) Time-series plot for the coast between Asfontilitis and Aegiali sites.

The LOS displacements estimated during this period (Figure 5) comply with the deformation pattern estimated during the previous period (Figure 3). Low-rate velocities, not exceeding 2 mm/y towards and away from the satellite, dominate in most parts of the island and the two islets. In the ascending track, the Hozoviotissa Monastery coast and the coast between Asfontilitis and Aegiali sites, are experiencing subsidence, but with lower velocity rates not exceeding 4 mm/y (Figure 6). While the results from both tracks for the majority of Amorgos agree, a significant difference is observed in the subsiding coasts of the ascending track. This shift in the direction of the deformation observed in the same coasts, for the descending track, is believed to be associated with horizontal movement contribution. Aiming to further investigate the type of motion detected in the LOS velocity plots, further analysis was performed, by decomposing the LOS velocity vectors to vertical and horizontal components. In Sentinel-1 deformation maps, the southeastern part of the island, to the East of Aegiali region, depicted with the black square in Figure 5 remains an uplifting area for both tracks, similarly to the 2003–2010 analysis, with a higher velocity rate in the ascending track and a maximum value of 4 mm/y.

## 5. LOS Displacements Decomposition

**Figure 7:**
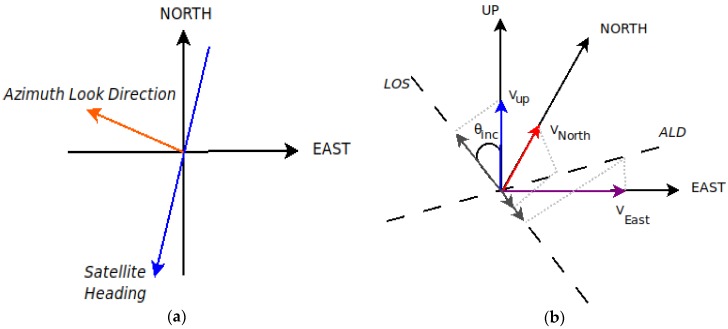
Geometry of the LOS velocity vector and its decomposition to North, East and Up components: (**a**) Satellite’s heading and Azimuth Look Direction for the descending satellite pass; (**b**) Projection of the Up, North and East velocities to the Line Of Sight (LOS) direction for the ascending satellite pass. Modified by: [38].

SAR interferometry estimates velocities in the Line-Of-Sight direction, therefore displacements refer to a direction away from or towards the satellite. InSAR sensitivity is higher in the vertical component of the movement and minimal to the north component [37]. This phenomenon is due to the near polar orbits and the small incidence angles of SAR satellites. Figure 7 shows the geometry of the satellite’s heading and Azimuth Look Direction (ALD) for the descending satellite pass, as well as the decomposition of the Up, East and North motion components to the Line Of Sight direction. The LOS velocities are the projection of 3D deformation on the line of sight direction (Equation (1)). The Azimuth Look Direction (ALD) comprises information about the E-W and N-S motion components and is always perpendicular to the satellite heading.

(1) $$v_{LOS}=v_{Up}\;+\;v_{East}+v_{North}$$

We performed a decomposition of the LOS velocities, of the ascending no.29 and descending no.36 Sentinel-1 imaging geometries, to the vertical and horizontal components in the descending look direction. The analysis is based on the hypothesis that a short distance between the PS scatterers reflects a correlation in deformation [39]. Following the methodology proposed by [40], for ERS and ENVISAT sensors and adjusting it accordingly for Sentinel-1 data, initially we formed a grid that includes the area of interest. There exist several methods for data downsampling, including uniform sampling [41], gradient-based quadtree sampling [42,43], resolution-based quadtree sampling [44,45]. A uniform downsampling can be adopted for subduction zone events or other deep sources of deformation [41,44]. On the other hand, a quadtree grid would be more reasonable for deformation sources that break the surface (e.g., areas near where a fault ruptures the surface) [41,44]. Gradient-based quadtree sampling creates grid cells of different sizes based on the displacement gradients, though sampled data may include noise, atmosphere and orbit errors. Resolution-based quadtree sampling forms a grid that is denser in areas near the deformation, while the values of resolution threshold and regularization parameters may result in data in non-deforming areas.

For estimating vertical and horizontal motion components to the whole extent of the island, the use of a uniform grid is well applied in our case study, since the deformation pattern is smooth and the averaging of the LOS velocities, do not result to a significant loss of the deformation signal [41]. To ensure the preservation of information relative to deformation phenomena, we performed several trials of decomposition with different grid cell sizes, and we finally opted for a patch size of 50 × 50 m. The selected grid cell size introduced a downsampling of the final displacements, yet we maintained this cell size, as there was no loss of information in the areas of special interest that needed further investigation.

The shape of the island required a constant control of the patches, in every step of the analysis, in order to exclude those with zero PS scatterers. The center of each cell is calculated, and a spline interpolation is used to estimate the look-angle of the center from the corresponding values of the PS pixels included in each cell. Within each patch an averaging of the LOS velocities is performed. For the heading angle rates, we used an average value for all pixels, which is around 350° for the ascending track and 190° for the descending track. For decomposing the LOS velocities to vertical and horizontal displacements, the system of Equations (2) and (3) for the descending azimuth look angle direction [37], was solved for every common scatterer between ascending and descending tracks. Only patches containing PS scatterers in both ascending and descending imaging geometries were included in the decomposition analysis.

(2) $$\left(\begin{array}{c} d_{LOS}^{asc}\\ d_{LOS}^{desc} \end{array}\right)=A\cdot \left(\begin{array}{c} d_{Up}\\ d_{ALD} \end{array}\right)$$

(3) $$A=\left(\begin{array}{cc} cos\;\left(\theta^{asc}\right) & \frac{sin\;\left(\theta^{asc}\right)}{cos\;\left(\Delta \alpha \right)}\\ cos\;\left(\theta^{desc}\right) & sin\;\left(\theta^{desc}\right) \end{array}\right)$$

where $d_{LOS}^{asc}$ is the LOS deformation of the ascending satellite pass, $d_{LOS}^{desc}$ is the LOS deformation of the descending satellite pass, $d_{Up}$ is the vertical velocity, $d_{ALD}$ is the projection of horizontal deformation in the descending azimuth look direction, $\theta^{desc}$ is the incidence angle of the descending satellite pass, $\theta^{asc}$ is the incidence angle of the ascending satellite pass, Δα is the satellite heading difference between ascending and descending pass.

Due to the limitation of one satellite pass for the Envisat data, an accurate comparison between the two periods is unfeasible. Though, we converted the LOS displacements from the 2003–2010 period, to vertical displacements using the Equation (4).

(4) $$v_{up}=\frac{v_{LOS}}{cos\;\left(\theta \right)}$$

where for every scatterer $v_{up}$ is the vertical velocity, θ is the incidence angle and $v_{LOS}$ is the line of sight velocity.

**Figure 8:**
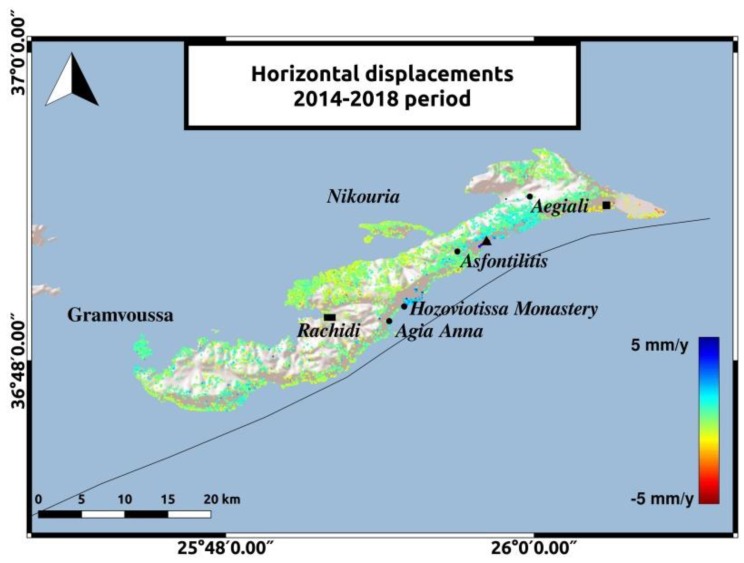
Horizontal displacements for the 2014–2019 period. The reference area near Rachidi is marked with the black rectangle. The black triangle represents the area between Asfontilitis and Aegiali sites and the black square represents the coast to the East of Aegiali.

**Figure 9:**
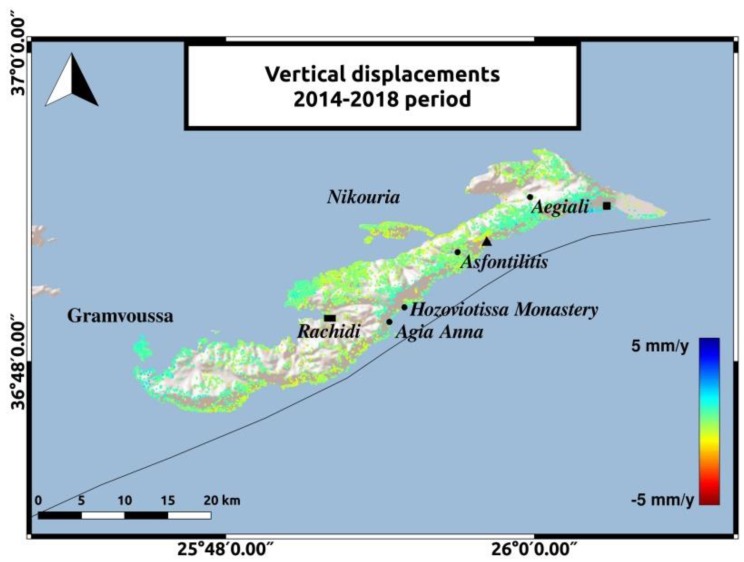
Vertical displacements for the 2014–2019 period. The reference area near Rachidi is marked with the black rectangle. The black triangle represents the area between Asfontilitis and Aegiali sites and the black square represents the coast to the East of Aegiali.

The decomposition to vertical and horizontal velocities adds some information about ground movements on the island. During both time periods, low rate displacements are observed in most parts of Amorgos. The two most significant observations will be mentioned. Horizontal motion in Hozoviotissa Monastery coast and uplift in the southeastern coast of Amorgos, to the East of Aegiali region, depicted with the black square in Figure 8 and Figure 9. The LOS displacements in Hozoviotissa Monastery region, as mapped in Figure 5 and Figure 6, are opposite between the two different satellite passes, and thus horizontal movement is more likely to occur. ALD velocities at this region and in the coast between Asfontilitis and Aegiali are positive, with a maximum value of 5 mm/y, indicating a westward trend that might be associated with strike-slip components, following the interpretation of previous studies [2] for the main structures on Amorgos island. Another significant observation is the vertical displacements in the southeastern coast of Amorgos, with a maximum value of 4 mm/y. According to previous studies [2,16], the southeastern coast is characterized by high relief, normal faulting and uplift. Figure 8 and Figure 9 present the horizontal and vertical velocities for Sentinel-1 data, respectively.

**Figure 10:**
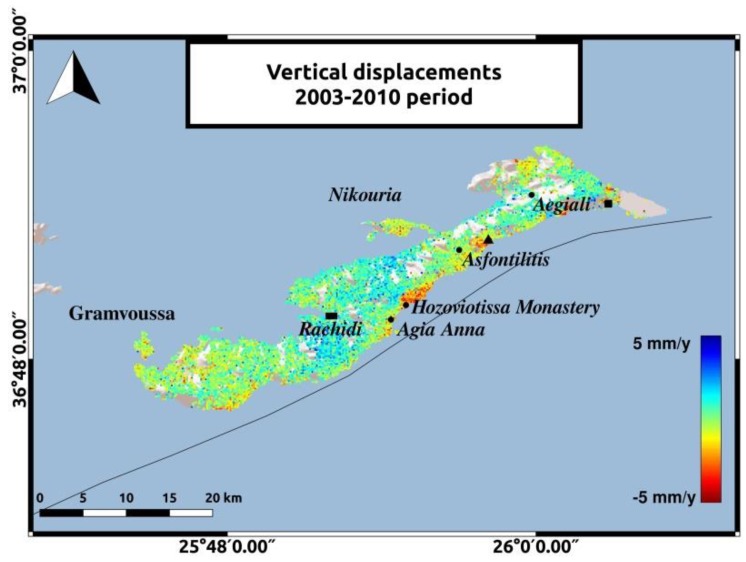
Vertical displacements for the 2003–2010 period for the ascending track no.57. The reference area near Rachidi is marked with the black rectangle. The black triangle represents the area between Asfontilitis and Aegiali sites and the black square represents the coast to the East of Aegiali.

The vertical velocities for Envisat data are mapped in Figure 10. Although, the comparison between the two periods is unfeasible, we can obtain some information about the displacement field for the period from 2003 to 2010. We observe negative vertical velocities with a maximum value of −5 mm/y in Hozoviotissa region and positive velocity rates of 4 mm/y in the southeastern coast of Amorgos. The use of leveling data and GPS measurements, along with the InSAR results, could provide a better connection of these values with tectonic processes on the island.

## 6. Discussion

Time-series analysis of Envisat and Sentinel-1 satellite data was performed to study ground deformation in Amorgos island, from 2003 to 2019. Multi-temporal interferometry (MTI) using PS and SBAS techniques, as well as the combination of the above methods, was implemented in Envisat data. Line of sight velocities for Sentinel-1 images were estimated, using the PSI technique. For the 2014–2019 period, we combined observations from different imaging geometries to decompose the LOS velocities to horizontal and vertical components. Horizontal motion is observed in Hozoviotissa Monastery area. In addition, we concluded that the southeastern coast of Amorgos is uplifted. The estimation of vertical velocities for Envisat data indicates subsidence in Hozoviotissa area and uplift on the southeastern coast of the island.

These results are consistent with the neotectonic observations performed in Amorgos [2] and the focal mechanisms of the 1956 large earthquake which show both normal and strike-slip components [2,7]. Uplift realized in southeastern Amorgos coast very likely is the gradual uplift of the footwall domain of the neotectonic fault dominating the area. This interpretation is consistent with the results of other authors [16].

On the other hand, subsidence in the Hozoviotissa area could be associated with the gradual subsidence of the hanging wall domain of the neotectonic fault. These upper-crust movements may reflect post-event long-term relaxation, which has been recognized to be caused by large earthquakes as redistribution of displacement, strain, and stress (e.g., [46,47,48,49]).

It is worthwhile mentioning that this is a first attempt to attain deformation monitoring in Amorgos island with the use of geodetic observations. Time-series analysis of InSAR data has proven to be a very promising tool to monitor ground deformation phenomena. In the present study, the estimated SAR displacements verified the results of past tectonic studies and were associated with tectonic processes on the island. Yet, the combination of SAR and GPS observations on the island is proposed as future work to increase the spatial, as well as temporal resolution, and contribute to a better validation of the estimated SAR displacements.

## Figures and Tables

**Table 1:** Data information.

	Sensors		
	Envisat Asar	Sentinel	
Number of scenes	24	54	70
Relative orbit	57	36	29
Time interval	2003–2010	2014–2019	2014–2019
Swath type	I2	n/a	n/a
Sensor’s pass	Ascending	Descending	Ascending
SAR mode	n/a	Interferometric Wide swath (IW)	Interferometric Wide swath (IW)

## Appendix A

This Annex includes the baseline plots of the interferometric stacks used for the time-series analysis in Amorgos island from 2003 to 2019.
